# Operative Surgery of the Eye

**Published:** 1834

**Authors:** 


### II.—OPERATIVE SURGERY OF THE EYE.

In a country where so few medical gentlemen devote themselves to the Operative Surgery of the Eye, it may be pardoned in one who has made some special preparation, by the importation of books and instruments, for that department of the profession, to make it known to his brethren. The EDITOR of this Journal, would, therefore, respectfully inform such of his brethren in the Valley of the Mississippi, as do not operate in diseases of the eye, that he has paid considerable attention to that part of surgery, and is prepared with the requisite instruments, for every operation which is usually performed on that organ.

Those which he has had occasion to perform are extirpation of the eyeball and of encysted tumours from the orbit; the removal of ectropion and entropion; petyrgium; fistula lachrymalis; cataract, by laceration, depression, projection into the anterior chamber, and extraction; staphyloma, artificial pupil, &c. &c.

